# Pediatric thyroid cancer presenting aggressive pathologic characteristics shows persistently high rates of recurrence during the past 35 years in Korea

**DOI:** 10.1186/1687-9856-2015-S1-P100

**Published:** 2015-04-28

**Authors:** Young Ah Lee, Hae Woon Jung, Hwa Young Kim, Hoonsung Choi, Choong Ho Shin, Sei Won Yang, Young Joo Park

**Affiliations:** 1 Division of Endocrinology and Metabolism, Department of Pediatrics, Seoul National University College of Medicine, Seoul, Korea; 2 Division of Endocrinology and Metabolism, Department of Internal Medicine, Seoul National University College of Medicine, Seoul, Korea

## Objective

Clinicopathological characteristics at diagnosis and long-term outcomes of pediatric thyroid cancer were analyzed. Predictors for poor outcome or recurrence were investigated among pediatric papillary thyroid cancer (PTC) patients. Whether young age at diagnosis (<20 years) was independently predictive for recurrence was investigated among PTC patients of all ages.

## Methods

We evaluated 153 patients (28males) diagnosed younger than 20 years old, managed during 1980 through 2013 (median 7.0 years of duration). Good or poor outcome (persistence or recurrence) was analyzed in 126 patients followed for at least 12 months. Predictors for recurrence were analyzed among 108 pediatric PTC patients. Adult PTC patients (n = 3093) were finally included in Cox proportional hazards models to find predictors for recur-free survival among PTC patients of all ages.

## Results

At the time of diagnosis [papillary (86.9%), follicular (7.9%), medullary (3.9%) and poorly differentiated (1.3%)], 38.6% of multiplicity, 57.8% of extrathyroidal extension, and 66.7% of lymph node (LN) and 13.9% of lung metastasis were found. The proportions of PTC, multiplicity, extrathyroidal extension, LN metastasis (all for P< 0.05), and distant metastasis (P< 0.001) were significantly higher in younger patients at diagnosis. Forty-four of 126 patients (34.9%) showed poor outcome. Recurrence rates at 5 and 10 years were 13.9% and 33.8% respectively. In an analysis of 108 pediatric PTC patients, the poor outcome group showed larger tumors (2.7cm vs. 2.0cm), higher rates of multiplicity (66.7% vs. 30.6%) and distant metastasis (27.0% vs. 7.7%) than the good outcome group (all P< 0.01). After adjusting for sex, age at diagnosis, primary tumor size and LN metastasis, both multiplicity and primary tumor ≥4cm (all P< 0.01) were independent predictors for recurrence among pediatric PTC patients. Male sex, multiplicity, LN metastasis (all P< 0.001), primary tumor ≥4cm (vs. 1.0-1.9cm), and extrathyroidal extension (P< 0.01 for both) rather than age at diagnosis (<20, 20-39, 40-59, ≥60 years) were independent predictors for a poor recur-free survival among PTC patients of all ages.

## Conclusions

Pediatric thyroid cancer presented aggressive pathologic characteristics and showed persistently high rates of recurrence during the past 35 years in Korea. The younger onset, the more aggressive was the pathologic presentation. Among pediatric PTC patients, large tumors (≥4cm) and multiplicity were predictive for recurrence. Among PTC patients of all ages, aggressive pathologic presentations at a young age rather than young age itself were decisive factors for recurrence.

